# Inhaled Nitric Oxide Treatment for Aneurysmal SAH Patients With Delayed Cerebral Ischemia

**DOI:** 10.3389/fneur.2022.817072

**Published:** 2022-02-18

**Authors:** Christian Fung, Werner J. Z'Graggen, Stephan M. Jakob, Jan Gralla, Matthias Haenggi, Hans-Ulrich Rothen, Pasquale Mordasini, Michael Lensch, Nicole Söll, Nicole Terpolilli, Sergej Feiler, Markus F. Oertel, Andreas Raabe, Nikolaus Plesnila, Jukka Takala, Jürgen Beck

**Affiliations:** ^1^Department of Neurosurgery, Medical Center, University of Freiburg, Freiburg, Germany; ^2^Department of Neurosurgery, Inselspital, Bern University Hospital, University of Bern, Bern, Switzerland; ^3^Department of Intensive Care Medicine, Inselspital, Bern University Hospital, University of Bern, Bern, Switzerland; ^4^Department of Diagnostic and Interventional Neuroradiology, Inselspital, Bern University Hospital, University of Bern, Bern, Switzerland; ^5^Institute for Stroke and Dementia Research (ISD), Munich University Hospital, Munich, Germany; ^6^Department of Neurosurgery, Munich University Hospital, Munich, Germany; ^7^Department of Neurosurgery, University Hospital Zurich, Zurich, Switzerland

**Keywords:** subarachnoid hemorrhage, vasospasm, INO, hypoperfusion, aneurysm, ischemia

## Abstract

**Background:**

We demonstrated experimentally that inhaled nitric oxide (iNO) dilates hypoperfused arterioles, increases tissue perfusion, and improves neurological outcome following subarachnoid hemorrhage (SAH) in mice. We performed a prospective pilot study to evaluate iNO in patients with delayed cerebral ischemia after SAH.

**Methods:**

SAH patients with delayed cerebral ischemia and hypoperfusion despite conservative treatment were included. iNO was administered at a maximum dose of 40 ppm. The response to iNO was considered positive if: cerebral artery diameter increased by 10% in digital subtraction angiography (DSA), or tissue oxygen partial pressure (PtiO_2_) increased by > 5 mmHg, or transcranial doppler (TCD) values decreased more than 30 cm/sec, or mean transit time (MTT) decreased below 6.5 secs in CT perfusion (CTP). Patient outcome was assessed at 6 months with the modified Rankin Scale (mRS).

**Results:**

Seven patients were enrolled between February 2013 and September 2016. Median duration of iNO administration was 23 h. The primary endpoint was reached in all patients (five out of 17 DSA examinations, 19 out of 29 PtiO_2_ time points, nine out of 26 TCD examinations, three out of five CTP examinations). No adverse events necessitating the cessation of iNO were observed. At 6 months, three patients presented with a mRS score of 0, one patient each with an mRS score of 2 and 3, and two patients had died.

**Conclusion:**

Administration of iNO in SAH patients is safe. These results call for a larger prospective evaluation.

## Introduction

Despite advances in emergency medicine, neurocritical care, and aneurysm occlusion techniques, aneurysmal subarachnoid hemorrhage (aSAH) remains a rare but severe subtype of stroke with high mortality and poor outcome (1, 2). Delayed cerebral ischaemia (DCI) is thought to be the major reason for poor outcome in survivors of aSAH (2). Up to this day and despite decades of experimental and clinical research, causes and pathomechanisms of DCI are still not completely clear and most probably multifaceted (3). Cerebrovascular spasm (CVS), a narrowing of cerebral arteries thought to be caused by blood breakdown products, usually peaks between day 5 and 14 after hemorrhage and can be observed in up to 88% of patients with severe aSAH (4). While it is still not completely understood whether angiographic CVS is an epiphenomenon or symptom of other mechanisms or a pathomechanism directly causing DCI, it is thought to relevantly contribute to DCI. Despite advances in its detection and treatment, 20–40% of CVS patients experience cerebral ischaemia, which is associated with an adverse outcome (5). So far, there is no established therapy to improve cerebral perfusion and thus avoid tissue ischaemia and infarct formation. In patients with critical cerebral hypoperfusion without established cerebral infarction, rescue therapies such as induced hypertension, intra-arterial application of vasodilators, or angioplasty are used to improve cerebral perfusion. The efficacy of such interventions is not proven, their effects are temporary at best, and adverse effects are possible, including exposure to radiation (6–8).

As early as the 1980ies, depletion of nitric oxide (NO), a potent endogenous vasodilator and mediator, was recognized to be heavily involved in the pathogenesis of delayed cerebral ischemia after SAH (9–11). Up to now there are, however, no established clinical tools to replenish NO to the cerebral circulation. In series of translational experimental studies using animal models for ischaemic stroke, traumatic brain injury, and SAH we demonstrated that inhaled nitric oxide (iNO) selectively dilates cerebral arteries and arterioles in hypoperfused brain tissue (12–15). After SAH in mice, iNO significantly reduced the number and severity of SAH-induced microvasospasm, thereby improving cerebral perfusion, survival, and functional outcome. Systemic blood pressure was not affected by iNO, which is a major advantage over other vasodilating treatment strategies (15, 16). In contrast to many other therapeutics evaluated in experimental studies, iNO already has regulatory approval for human use from the United States Food and Drug Administration and the European Medicines Agency in the treatment of several pulmonary pathologies (e.g., respiratory distress syndrome, pulmonary hypertension) in infants (17–20) and in adults (21, 22) and has a good safety record of use in humans since the 1990s. The efficacy of iNO was thought to be limited to the lungs but, given the promising results of the experimental studies and its safety record, we hypothesized that iNO may improve cerebral perfusion in patients with aSAH. We therefore performed a prospective pilot study evaluating the effects of iNO on cerebral perfusion in patients with delayed cerebral ischemia after aSAH.

## Methods

### Inclusion Criteria

Patients were included in the prospective study at the Department of Neurosurgery of the University Hospital Bern, Switzerland. Approval was obtained from the local ethics committee (Kantonale Ethikkommission, Bern, Switzerland, KEK ID 019/10) and Swissmedic (Bern, Switzerland, ID 2012 DR 2135), and the study conformed to the Declaration of Helsinki. Only patients with persisting delayed cerebral ischemia after standard therapy options had been exhausted were eligible. Inclusion criteria were aSAH of all severities [World Federation of Neurosurgeons (WFNS) score I–V], aneurysm treated by either surgical clipping or endovascular coiling, age between 18 and 80 years, proven CVS, delayed cerebral ischemia and neurological deficit despite treatment (oral nimodipine, induced hypertension, hypervolaemia, central venous pressure > 6 mmHg), a negative pregnancy test in women, and signed informed consent from the next of kin and an independent physician.

### Exclusion Criteria

Exclusion criteria were (i) unsecured aneurysm, (ii) cerebral infarction on imaging in the downstream brain parenchyma of spastic vessel, (iii) cerebral herniation, (iv) intracranial pressure > 25 mmHg, (v) pregnancy, and (vi) mean arterial pressure ≤ 90 mmHg despite catecholamines.

### Definition of Vasospasm

Cerebral vasospasm was diagnosed by either (i) digital subtraction angiography (DSA) (vessel narrowing >66%), (ii) transcranial Doppler (TCD) sonography measurement (mean flow velocity > 150 cm/s and/or Lindegaard index > 3, and/or increase of TCD velocity of more than 50 cm/s within 24 h), or (iii) cranial computed tomography (CT) angiography (vessel narrowing > 66%). The vessel most affected by the CVS in DSA and which was responsible for the neurological deficit was defined as the index vessel. Cerebral hypoperfusion/ delayed cerebral ischemia was diagnosed by either (i) newly occurring neurological deficit (drop in Glasgow coma scale (GCS) of ≥ 1 point or an increase in the National Institutes of Health Stroke Scale score (NIHSS) by ≥ 2 points), (ii) reduced intraparenchymal tissue oxygen partial pressure (PtiO_2_) < 10 mmHg, (iii) mean transit time (MTT) > 6.5 s in CTP, or (iv) cerebral circulation time > 5 s in DSA imaging.

CTP was assessed at three levels: section Introduction at the level of the basal ganglia, section Methods at the level of the lateral ventricle, and section Results above the lateral ventricle. On section Introduction we defined 12 regions of interest (ROIs) and on sections Methods and Results, 10 ROIs each. MTT was assessed for every ROI resulting in 32 ROIs per CTP measurement. We then calculated the sum of ROIs with impaired perfusion (MTT > 6.5 s) for each measurement.

### Patient Monitoring

All patients were intubated, mechanically ventilated and sedated with either propofol or midazolam, and received fentanyl for analgesia as well as for muscle relaxation during angiography. Hemodynamics were monitored continuously using intra-arterial and pulmonary artery catheters, including continual cardiac output and mixed venous oxygen saturation. Pulse oximetry and end-tidal CO_2_ were also continuously monitored. In addition, brain tissue oxygen partial pressure (PtiO_2_) (Integra Licox^®^, Integra LifeSciences, Saint Priest, France) and intracranial pressure (ICP) using an external ventricular drain catheter were measured. PtiO_2_ probes were placed into the white matter of the frontal lobe.

### Application of iNO

We applied inhaled nitric oxide (INOmax^®^, Linde France, Porcheville, France) as an additive to the ventilation mixture in intubated patients using a specialized delivery system (INOmax^®^ DS_IR_ Plus, Linde Healthcare, Germany) and ventilator (Servo-I, Marquet Critical Care, Solna, Sweden). After assessing all baseline parameters, iNO was started at a dose of 1 parts per million (ppm) and increased stepwise to 2, 5, 12, 25 ppm, until a maximum dose of 40 ppm was reached. Each increase of iNO dose was followed by a 10-min monitoring period and a DSA examination. After reaching the highest effective dose of iNO or the maximum of 40 ppm we performed another DSA. iNO was continued until normalization of CVS or for a maximum period of 5 days. During iNO treatment a DSA was performed every 24 h, followed by a decrease of iNO to the next-lower level. If tapering the iNO concentration was associated with increasing vasospasm, iNO was increased again to the last effective dosage. For cessation of iNO administration, dosage was tapered every 30 mins using the same dosage steps as for initiation of iNO treatment. If the duration of iNO treatment was more than 32 h, tapering intervals were prolonged to 4 h. Cessation of iNO was followed by a DSA.

### Evaluation of Efficacy

The primary endpoint of this study was improvement of severe vasospasm, as indicated by any of the following prespecified criteria:

DSA: a > 10% increase in diameter of the vasospastic target vessel compared to baseline (at the end of the titration phase and every 24 h under iNO treatment);

PtiO_2_: an increase of more than 5 mmHg with constant fraction of inspired oxygen (FiO_2_) (at the end of the titration phase, and 4, 12, 24, and 48 h after initiation of iNO);

TCD: a decrease of more than 30 cm/s (at the end of the titration phase, and 4, 12, 24, and 48 h after initiation of iNO);

CTP: a reduction in the number of ROIs with impaired perfusion (MTT > 6.5 s).

### Evaluation of iNO Safety

Methaemoglobin levels were assessed every 8 h. A level below 5% was considered normal in non-smokers and < 10% in smokers. Systemic blood pressure was continuously monitored, and vasopressors increased to keep blood pressure at baseline level, if necessary. If baseline systemic arterial pressure could not be maintained by increasing doses of catecholamines, iNO was discontinued. Since it may suppress intrinsic NO production, abrupt cessation of iNO may cause pulmonary arterial hypertension. If there was a clinically relevant increase in pulmonary artery pressure, as defined by the treating physician, iNO was increased to the previous levels and then decreased with longer time intervals between each reduction step. Creatinine was checked daily to monitor kidney function. Inhaled NO may increase intracranial pressure, so patients were monitored using invasive ICP measurements.

Pre-defined safety parameters that triggered premature cessation of iNO treatment were: ICP > 25 mmHg, decrease of PtiO_2_ > 5 mmHg, increase of CVS as assessed by DSA, mean arterial pressure (MAP) < 60 mmHg and cerebral perfusion pressure (CPP) < 50 mmHg, uncontrollable systemic hypertension, renal insufficiency indicated by increase of serum creatinine > 50% or absolute 120 μmol/l, NO_2_ in respiratory gas mix of > 0.5 ppm, methaemoglobin > 5% (> 10% in smokers) and/or coagulation abnormalities with blood loss (hemoglobin < 70g/l, thrombocyte count < 70,000G/l, international normalized ratio (INR) > 1.8, partial thromboplastin time (pTT) > 40 secs or clinical signs of an increased bleeding tendency (e.g. bruises, gum bleeding, bleeding from surgical wounds).

### Follow-Up

Follow up examination was performed at 12 weeks and 6 months after SAH at the outpatient clinic. It included assessment of NIHSS, Mini Mental State (MMS) test, and modified Rankin Scale (mRS). Favorable outcome was defined as mRS score ≤ 3. As the incidence of cerebral infarctions has been shown to be a strong predictor for outcome (23) we performed a CT scan at a minimum of 12 weeks after ictus to assess ischaemic events. The volume of ischaemic tissue was quantified in cubic centimeters (cm^3^) using volumetry (iPlan, Brainlab, Munich, Germany).

### Statistics

Owing to the small number of patients, statistical analysis was confined to descriptive statistics. In addition to absolute values, vessel diameter was calculated as percentage change from baseline. Continuous variables are given as mean (IQR) and range.

## Results

Between February 2013 and September 2016, we enrolled seven patients with delayed cerebral ischemia into the study, five females and two males. Mean age was 51, 7 years (Table 1). One patient was admitted with a World Federation of Neurosurgical Societies (WFNS) grade 1, three with WFNS 2, and three with WFNS grade 4 SAH. Aneurysm closure was achieved by coiling in six patients and by microsurgical clipping in one patient. Aneurysms were located on the anterior communicating artery (*n* = 6) or posterior communicating artery (*n* = 1). Prior to their inclusion in this study all patients were severely affected by delayed cerebral ischemia despite maximum treatment and showed clinical deterioration: patient 1 presented new hemiparesis (M4, left arm and M0, left leg) followed by a drop in GCS from 15 to 5. Patient 2 had a decrease in GCS from 15 to 14 and new hemiparesis (M4, left). Patient 3's GCS dropped from 14 to 11 and paresis of the left arm was noted (M4). Patient 4 showed a decrease in GCS from 12 to 10 and progressive hemiparesis (right arm M2, right leg M3). Patient 5 suffered progressive paresis (both legs M2) and showed a drop in GCS from 14 to 13. In patient 6 GCS decreased from 15 to 12 and in patient 7 from 14 to 8 points.

**Table 1:** Demographics and outcome.

**Patient no**.	**Sex**	**Age**	**WFNS**	**Aneurysm**	**Treatment**	**Target vessel**	**Side**	**Duration of iNO (min)**	**GCS before initiation of iNO**	**NIHSS before initiation of iNO**	**3-month follow-up**			**6-month follow-up**			**Volume of ischaemic area (cm^**3**^)**
											**mRS**	**NIHSS**	**MMS**	**mRS**	**NIHSS**	**MMS**	
1	M	39	2	ACOM	Coiling	M1	Right	3,226	5	24	6	NA	NA	6	NA	NA	657
2	F	53	1	ACOM	Coiling	M1	Right	1,213	14	6	1	2	30	2	2	^a^	111
3	F	62	2	ACOM	Clipping	MCA	Right	1,401	11	8	0	0	28	0	0	25	0
4	F	46	4	PCOM	Coiling	M1	Left	1,121	10	13	2	6	3	3	6	3	49
5	F	35	4	ACOM	Coiling	A1	Right	4,160	13	16	6	NA	NA	6	NA	NA	148
6	F	69	2	ACOM	Coiling	A1	Right	1,379	14	3	2	0	29	0	0	30	0
7	M	58	4	ACOM	Coiling	A2	Right	1,310	^b^	^b^	1	0	27	0	0	30	0

a *Lost to follow-up*,

b *Intubated and analgosedated*.

*WFNS, World Federation of Neurosurgeons; iNO, inhaled nitric oxide; GCS, Glasgow coma scale; NIHSS, National Institutes of Health Stroke Scale; ACOM, anterior communicating artery; MMS, Mini Mental State; mRS, modified Rankin scale*.

### Evaluation of iNO Efficacy

The response to iNO was considered positive in every patient included in the study (Table 2). Patient 1 reached an endpoint eight times, patient 2 four times, patient 3 seven times, patient 4 three times, patient 5 twice, patient 6 three times, and patient 7 nine times. The number of times a patient reached the combined primary endpoint was not associated with the duration of iNO treatment. One patient (patient 7) reached the endpoint in all four parameters tested (DSA, PtiO_2_, TCD, CTP); 2 patients met three endpoint criteria (patient 1: PtiO_2_, TCD, CTP; patient 3: DSA, PtiO_2_, TCD), two patients met two criteria (patient 2: DSA, PtiO_2_; patient 6: PtiO_2_, CTP), and two patients reached only the PtiO_2_ criterion.

**Table 2:** Efficacy.

	**Target vessel diameter in DSA**				**PtiO_2_ (mmHg)**						**TCD (cm/sec)**						**CTP (no. of ROIs with MTT < 6.5)**		
**Patient No**.	**Baseline (mm)**	**After titration (% from baseline)**	**24 h (% from baseline)**	**48 h (% from baseline)**	**Baseline**	**After titration**	**4 h**	**12 h**	**24 h**	**48 h**	**Baseline**	**After titration**	**4 h**	**12 h**	**24 h**	**48 h**	**Baseline**	**Scan 1**	**Scan 2**
1	2.40	2.4 (0)	2.01 (−16.3)	2.1 (−12.5)	27.0	6.2	34.2	28.9	24.9	32.6	239	199	109	96	180	103	31	15	
2	1.6	1.8 (12.5)	0.9 (−43.8)	1 (−37.5)	8.0	10.1	15.7	21.4	22.1		89	147	120	135	139		32	8^a^	
3	0.96	1.64 (70.8)	2.14 (122.9)		30.7	46.4	47.6	38.5	45.7		91	57	94	81	90		31	15^b^	
4	1	0.9 (−10.0)	0.8 (−20.0)		20.7	27.3	…	27.4	26.4		NA	NA	NA	NA	NA		…	…	
5	0.88	0.95 (8.0)	0.9 (2.3)	0,73 (−17.0)	39.3	45.5	52.5	33.0	27.9	17.9	74	68	60	130	97	130	11	30	24
6	1.51	1.49 (−1.3)	1,5 (−0.7)		28.4	31.8	31.7	37.3	52.4		41	24	35	22	38		3	0	
7	0.67	0.92 (25.0)	1.61 (140.3)		19.8	24.6	26.6	30.4	38.3		140	113	66	46	59		17	13	

*DSA, digital subtraction angiography; PtiO_2_, brain tissue oxygen; TCD, transcranial Doppler; CTP, computed tomography perfusion; ROI, region of interest; MTT, mean transit time*;

a *CTP was performed one day after iNO cessation*;

b *CTP was performed 2 days after iNO cessation. Green color indicates that the respective endpoint was reached*.

All but one patient tolerated and subsequently received the maximum iNO dosage of 40 ppm. Median duration of iNO application was 23 h (range 18.7–69.3). Patient 1 showed decreasing PtiO_2_ values to a minimum of 6.2 mmHg of the right hemisphere during iNO titration until 40 ppm. iNO was reduced and continued at 2–5 ppm. PtiO_2_ values increased above baseline values within 25 mins.

### Digital Subtraction Angiography (DSA)

**Figure 1:**
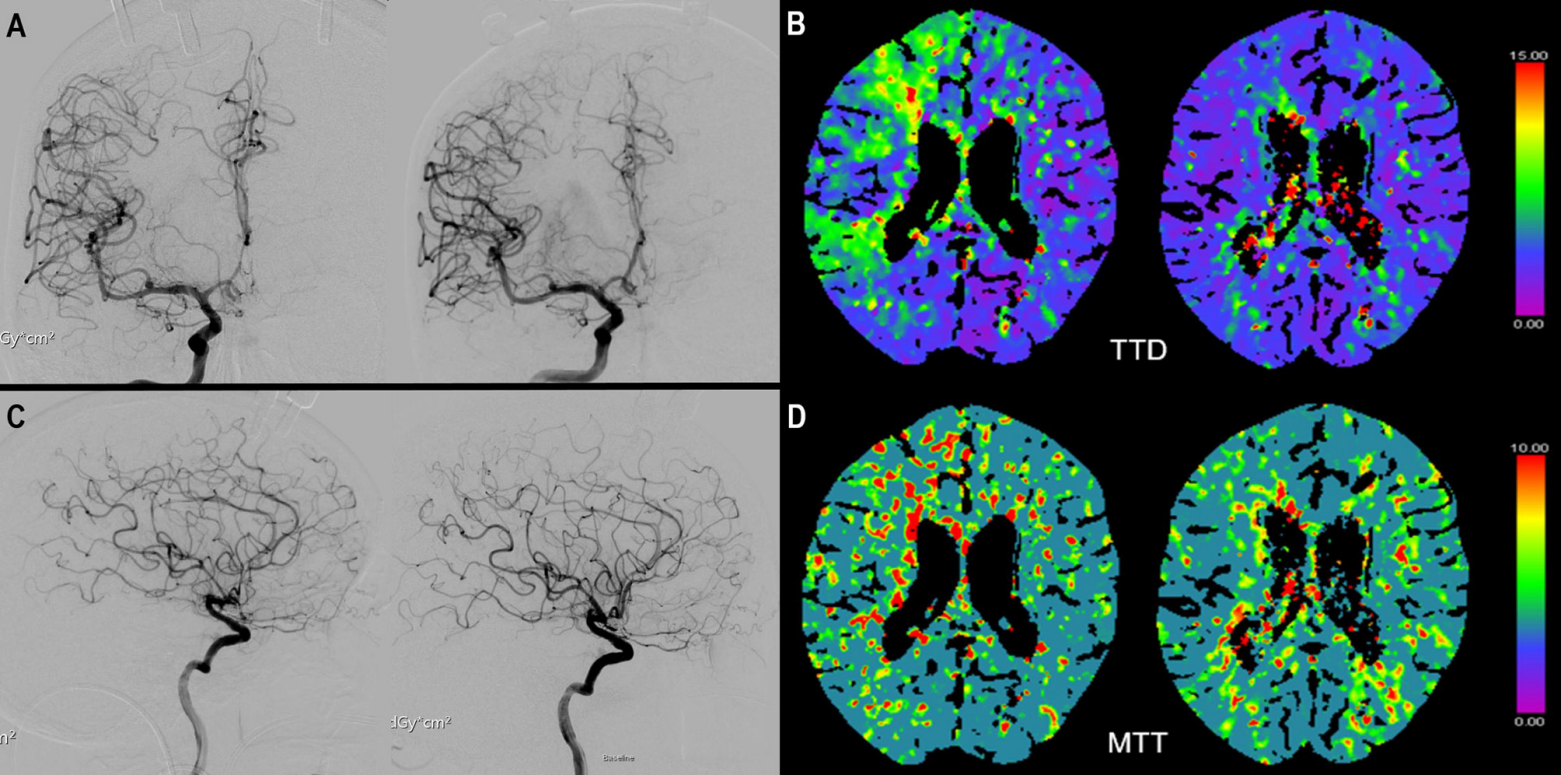
**(A)** ap view of a digital subtraction angiography (DSA) of the right internal carotid artery (ICA) before (left) and after (right) start of iNO application. Not the vasospastic vessels before initiation of iNO in the middle cerebral artery (MCA) and anterior cerebral artery territory (ACA). **(C)** shows the respective lateral view before (left) and (after) initiation of iNO. **(B)** (TTD) and **(D)** (MTT) show the corresponding CTP examination with perfusion deficits in the MCA and ACA territory and improvement after iNO therapy (right side).

Follow-up DSA after complete titration of iNO to the maximum dose of 40 ppm and 24 h after initiation of iNO, showed an increase of spastic vessel diameter >10% in the index vessel in three patients (patients 2, 3, and 7) and after 24 h in two patients (patients 3 and 7) (Figure 1). In three patients (patients 1, 2, and 5), we performed another DSA 48 h after initiation of iNO. None of these patients had any increase in vessel diameter at this time point. Patients 1, 4, 5, and 6 did not show a positive response to iNO treatment as measured by DSA at any time point.

### Tissue Oxygen Partial Pressure (PtiO_2_)

At baseline, cerebral oxygen partial pressure was below 20 mmHg in two patients (patients 2 and 7). Median PtiO_2_ values at baseline, after titration, and at 4, 12, and 24 h after initiation of iNO were 27.7 mmHg (range 8–39.3), 27.3 mmHg (range 6.2–46.4), 33.0 mmHg (range 15.7–52.5), 30.4 mmHg (range 21.4–38.5), and 27.9 mmHg (range 22.1–52.4), respectively. Immediately after titration to 40 ppm iNO, three of the seven patients (patients 3, 4, and 5) showed an improvement of PtiO_2_ of more than 5 mmHg. At 4, 12, and 24 h after initiation of iNO, five patients at each timepoint showed an improvement of PtiO_2_ of more than 5 mmHg, thereby reaching the primary endpoint. At 4 h after initiation of iNO, patients 1, 2, 3, 5, and 7; and at 12 and at 24 h after initiation, patients 2, 3, 4, 6, and 7 reached the primary endpoint. At 48 h after iNO initiation, one (patient 1) of two patients showed an increase > 5% (Table 2).

### Transcranial Doppler Assessment

One patient (patient 4) had no bony window for TCD assessments and was therefore excluded from TCD analysis. Median TCD values for the index vessel at the given timepoints (baseline, after titration, 4, 12, and 24 h after initiation of iNO) were 90 cm/s (range 41–239), 90.5 cm/s (range 24–199), 80 cm/s (range 35–120), 88.5 cm/s (range 22–135), and 93.5 cm/s (range 38–180), respectively. Two patients at each timepoint showed a decrease in TCD values > 30 cm/s compared to baseline measurement (after titration: patients 1 and 3, later timepoints: patients 1 and 7). At 48 h after initiation of iNO, one (patient 1) of two patients showed a decrease of TCD values of more than 30 cm/s compared to baseline.

### CT Perfusion

CTP measurements were available for six patients at baseline. Four of them had a subsequent CTP scan during iNO treatment. In three of these four patients perfusion improved during iNO treatment (Figure 1). The number of ROIs with MTT perfusion deficit decreased from 31 to 15 (patient 1), 3 to 0 (patient 6), and 17 to 13 (patient 7), respectively.

In patient 5 the number of critically hypoperfused ROIs increased from 11 to 30; in a subsequent CTP scan the number of MTT impaired ROIs in this patient had decreased again (to 24) but did not return to baseline. In the remaining two patients, the second CTP was performed not during, but 24 and 48 h after cessation of iNO. Also, in these patients, the number of critically hypoperfused ROIs was reduced compared to baseline from 32 to 8 (patient 2) and 31 to 15 (patient 3).

### Evaluation of iNO Safety

There were no safety concerns necessitating discontinuation of iNO treatment in any patients (Supplementary Table 1). In patient 1, iNO was reduced because of decreasing PtiO_2_ values but was continued at a lower dosage. The haemodynamic variables were maintained within 10% of their baseline values, but increased support with catecholamines was required by all patients. The median of individual mean arterial pressure during iNO application ranged from 113 mmHg (patient 2) to 151 mmHg (patient 5). Median increase of noradrenalin dose was 700 μg per hour (range 500–2,950), and in two patients additional dobutamine was started after initiation of iNO at a maximum dose of 20 mg per hour.

Median individual ICP values ranged from 7.0 mmHg (patient 2) to 15 mmHg (patients 6 and 7). Maximum ICP per patient during iNO application ranged from 16 (patient 4) to 19.8 mmHg (patient 5). ICP levels never exceeded 25 mmHg, the pre-defined stopping point.

Methaemoglobin levels remained below 5% in all patients at all timepoints. Kidney function, as assessed by creatinine levels, was unimpaired during the study. No adverse effects of iNO treatment on INR, thrombocyte count, or partial thrombin time were observed.

### Outcome

Two patients (patients 1 and 5) died during the acute phase of SAH (in-hospital mortality 29%), one of these during iNO administration, the other at a later time point. The patients died due to transtentorial herniation caused by extensive cerebral infarction. At 12 weeks, all surviving patients had a favorable outcome (mRS 2 or better); one patient presented no symptoms at all (mRS = 0), while two patients achieved a mRS score of 1 and two a score of 2. Only two patients had neurological deficits according to the NIHSS score (patient 2, NIHSS 2; patient 4, NIHSS 6). Four of five patients achieved normal results (27–30 points) in MMS assessment whereas the remaining patient had significant deficits. At 6 months, three patients were symptom-free (mRS 0), two of these patients had a better mRS score than at 12 weeks. In two patients, the mRS score had declined by one point (patient 2, from mRS 1 to mRS 2 and patient 4, from mRS 2 to mRS 3). These two patients were the only ones to present a relevant neurological deficit as assessed by NIHSS and MMS, and detectable cerebral infarction (ischaemic brain tissue volume 111 and 49 cm^3^), whereas the others showed nearly complete functional remission and no infarctions (Table 1).

Patient 1 presented the first small ischaemic demarcation after endovascular therapy. Further infarction was present on CT imaging at onset of iNO treatment and increased throughout iNO treatment to 657 cm^3^. In patient 2 the first ischaemic demarcation was shown on CT imaging 2 days after iNO cessation, with increasing infarct volume on follow-up CT 3 days later. In patient 4 the first ischaemic demarcation was seen 8 days after cessation of iNO, without further increase in size. In patient 5 the first ischaemic tissue was evident on CT imaging 2 days after onset of iNO treatment. Infarcts developed during iNO treatment and increased in size to 148 cm^3^ during the course of treatment.

## Discussion

Some years after introduction of iNO for the treatment of lung diseases, reports about its extrapulmonary effects started to emerge (24–27). Several investigators found evidence for the formation of bioactive NO compounds that deliver NO to extrapulmonary vascular beds (28–30). The first report that inhaled NO was transported to the cerebral vasculature in a murine model of ischaemic stroke appeared in 2012 (12). NO plays an important role in the pathophysiology of post-SAH brain damage and in the development of post-hemorrhagic ischaemia. Early on after SAH (from minutes to 72 h), NO concentrations are significantly reduced (31–33), most probably because of dysfunction of the endothelial NO-synthase (34), but there are numerous other pathomechanisms (NO scavenging by hemoglobin in the subarachnoid blood clot, endogenous nitric oxide synthase inhibitors, degeneration of perivascular nitrergic nerves, uncoupling of endothelial nitric oxide synthase, reactive oxygen species, or rho kinase activation) that add to a post-hemorrhagic NO-deficit (35–38). This local NO-deficit is thought to be one of the main factors leading to spasms in the cerebral microcirculation (15) and the early microcirculatory dysfunction (31–33, 39, 40) in experimental (41, 42) and clinical SAH (43, 44). NO depletion is also thought to contribute to post-hemorrhagic microthrombosis formation (45, 46), inflammatory changes (47), and spreading ischemia following cortical spreading depolarizations (CSDs) (35, 48, 49) which—in turn—further aggravate microcirculatory dysfunction and—thus—post-hemorrhagic ischemia after SAH. Furthermore, later on in the course of the disease (days after hemorrhage), NO is thought to be inactivated/scavenged by blood breakdown products and seems to be strongly linked to the development of delayed CVS (50–52). In experimental SAH (15), iNO reduced post-hemorrhagic micro-arterial constriction and microcirculatory dysfunction resulting in improved functional and structural outcome. Impaired autoregulation, another factor strongly implicated in the pathogenesis of post-SAH brain damage (53), was also shown to be positively influenced by iNO therapy in experimental SAH (54, 55) as was brain oedema formation (13, 15). NO inhalation therapy has previously been demonstrated to act anti-inflammatorily (26, 56, 57) and reduce platelet aggregation (58–61) in other disease models and may therefore act on many pathomechanisms associated with post-hemorrhagic brain damage beyond improvement of microcirculatory dysfunction. Also, cortical spreading depolarizations/ cortical spreading ischemia, which have been heavily implicated in the pathogenesis of DCI (62, 63), are positively influenced by increase of NO concentrations (64, 65), therefore amelioration of CSD induced ischemia may be another pathway of iNO mediated neuroprotection after SAH.

To the best of our knowledge, this is the first trial in humans evaluating the effect of iNO in patients with aSAH and severe (delayed) cerebral ischemia. We performed extensive cardiovascular and cerebrovascular monitoring including pulmonary catheter, repeated DSA of the brain, TCD, CTP, brain PtiO_2_, and ICP measurements. Due to this extensive monitoring each study patient required a significant amount of manpower. This in conjunction with the strict eligibility criteria has led to the low recruitment rate. All seven patients studied showed a positive response of reduction of cerebral ischemia during iNO application. Two patients died as a result of massive cerebral infarction. In one (patient 1) infarction occurred within the first 24 h after the onset of iNO treatment and the other (patient 5) had severe aggravation in the course. Outcome in the surviving patients was favorable (mRS ≤ 3) at both follow-up times (3 and 6 months after SAH). All subjects included in this study were severely affected by cerebral ischemia despite maximum medical treatment. Our results are descriptive and, though promising, do not allow conclusions on the efficacy of iNO to be drawn since no control group was included. Yet, in patients with delayed cerebral ischemia, morbidity and mortality is high and treatment options are limited to rescue therapies (66–69). In a recent study, reported a poor outcome (mRS 4–6) 1 year after SAH in 62% of patients that did not respond to medical therapy for symptomatic vasospasm (70). In our cohort of patients who did not respond to medical therapy, after application of iNO the percentage with a poor outcome was 29%. In these severely affected patients such a reduction of poor outcome is worthwhile.

The iNO treatment effect was most consistent in changes in intraparenchymal PtiO_2_: every patient reached the endpoint of a PtiO_2_ increase of more than 5 mmHg. In contrast, only three patients had an improvement as measured by TCD. This is in line with previous results from animal experiments showing cerebrovascular dilation induced by iNO predominantly in small vessels (12, 15), whereas the effect on larger vessels such as the basilar artery was short-lasting and not as pronounced as in the microcirculation (15). Angiography and TCD sonography may not be able to detect iNO-induced changes in the microcirculation. CTP scans can detect and monitor cerebral hypoperfusion in SAH patients (71–74) but since these scans were not done in all patients during iNO, the results should be interpreted with caution. Nevertheless, three of the four patients who had CTP scans during iNO had improved cerebral perfusion. In experimental brain injury, iNO effects were mainly evidenced in cerebral microvessels (12, 13, 15). In the present study, iNO effects were most reliably detected in brain oxygenation changes and CT perfusion studies, diagnostic tools that reflect microcirculatory perfusion; we therefore think it reasonable to assume that iNO mediated neuroprotection in humans may also act on the level of the cerebral microcirculation. Therefore, examinations like DSA or TCD might not record be the most suitable tools to detect iNO mediated changes, extended neuromonitoring including measurement of autoregulation status and occurrence of cortical spreading depolarizations may be advantageous in future investigations. In addition, delayed cerebral ischemia is a complex pathophysiological entity with angiographic vasospasm being only one of the factors involved (75, 76).

Since no previous data on iNO application in SAH patients were available, we used extensive cardiovascular and cerebrovascular monitoring for safety reasons and a large variety of established clinical diagnostical tools. In one patient the dosage of iNO had to be reduced owing to decreasing PtiO_2_ values. In the remaining patients no acute serious adverse reactions attributable to iNO were observed. This is consistent with the results of studies of iNO in lung and cardiovascular disease (19, 20, 22, 77–86). Vasodilation might have a negative impact on ICP and therefore also on cerebral perfusion pressure. However, with an external ventricular drain in place, ICP levels remained normal in all patients. Owing to ICP fluctuations in individual patients, ICP should be monitored and measures taken to control it during iNO application. In the clinical setting, systemic hypotension is always a concern with NO donors. Their use in (ischaemic) stroke has been considered to be contraindicated (87). Systemic hypotension and decreased cerebral perfusion pressure in patients with SAH and critical cerebral perfusion (88) would be particularly risky, counteracting any positive therapeutic effect. In studies in SAH patients, several investigators reported promising outcomes using NO-donating drugs in small clinical trials (89, 90). So far, however, no therapeutic strategy targeting this pathophysiological pathway has been successfully introduced into the standard treatment regime for SAH despite positive experimental results (91–96).

In our study, vasopressor dosage had to be increased in all patients to maintain MAP within the pre-defined target range. The increased need for vasopressors may be related to the effects of anesthesia and sedation rather than to iNO, and an increasing need for vasopressors is not uncommon in sedated patients with severe SAH (97). Cessation of iNO did not cause rebound pulmonary arterial hypertension or other undesirable haemodynamic effects in the present study. Nevertheless, the observed increase in catecholamine support emphasizes the need for close and comprehensive haemodynamic monitoring and timely interventions at the initiation, during, and after discontinuation of iNO administration.

This pilot study describes the safety of iNO in a very small number of highly selected patients and no conclusions on the general applicability of iNO in SAH patients are possible Notwithstanding the limitations of the present investigation, we believe that this pilot study provides a starting point for a translational, new, promising treatment approach for post-hemorrhagic brain damage that merits further evaluation in aneurysmal SAH patients.

## Data Availability Statement

The raw data supporting the conclusions of this article will be made available by the authors, without undue reservation.

## Ethics Statement

The studies involving human participants were reviewed and approved by Kantonale Ethikkommission, Bern, Switzerland, KEK ID 019/10 and Swissmedic (Bern, Switzerland, ID 2012 DR 2135). The patients/participants provided their written informed consent to participate in this study.

## Author Contributions

CF, WZ, SJ, JG, MH, H-UR, PM, ML, NS, NT, SF, MO, AR, NP, JT, and JB: data acquisition and review and editing of the manuscript. CF, NT, and JB: drafting of the manuscript. JB and WZ: study design. ML and NS: project administration. All authors contributed to the article and approved the submitted version.

## Conflict of Interest

The authors declare that the research was conducted in the absence of any commercial or financial relationships that could be construed as a potential conflict of interest.

## Publisher's Note

All claims expressed in this article are solely those of the authors and do not necessarily represent those of their affiliated organizations, or those of the publisher, the editors and the reviewers. Any product that may be evaluated in this article, or claim that may be made by its manufacturer, is not guaranteed or endorsed by the publisher.
