# Supplementary material for: Inhaled Nitric Oxide Treatment for Aneurysmal SAH Patients With Delayed Cerebral Ischemia
Source: Front Neurol. 2022 Feb 18;13:817072. doi: 10.3389/fneur.2022.817072 (PMC8894247; doi:10.3389/fneur.2022.817072)
Supplement: Supplementary file 1 [file Table_1.DOCX]

**Supplementary Material**

**Table 1: Safety**

|  | 1 | 2 | 3 | 4 | 5 | 6 | 7 |
| --- | --- | --- | --- | --- | --- | --- | --- |
| **Median MAP** (mmHg)(range) | 133 (119–152) | 113 (102–125) | 129 (120–138) | 124 (114–136) | 151 (129–180) | 129 (114–153) | 143 (133–153) |
| Met-Hb | 0·9 (0·7–1·5) | 0·7 (0·7–0·7) | 3·5 (2·4–3·6) | 1·8 (1·55–1·95) | 1·9 (1·8–2.0) | 2·6 (1·9–2·8) | 2·65 (2·0–2·78) |
| **Catecholamines** |  |  |  |  |  |  |  |
| Noradrenalin (µg per hour) |  |  |  |  |  |  |  |
| Before iNO | 400 | 1000 | 800 | 900 | 240 | 300 | 1050 |
| Maximum during iNO | 900 | 1700 | 1450 | 1700 | 800 | 1380 | 4000 |
| Dobutrex (mg per hour) |  |  |  |  |  |  |  |
| Before iNO | 0 | 0 | 0 | 0 | 0 | 0 | 20 |
| Maximum during iNO | 0 | 20 | 0 | 9 | 0 | 0 | 20 |
| **ICP** absolute values (mmHg) |  |  |  |  |  |  |  |
| Before iNO | 11·9 | 17 | 4 | 13 | 16·3 | 11 | 14·2 |
| Minimum during iNO | 3·0 | 0·6 | 1·6 | 8·0 | 6·9 | 4·0 | 9·6 |
| Maximum during iNO | 17 | 18 | 18·4 | 16 | 19·8 | 17 | 17·5 |
| Median ICP (IQR) | 10·9 (8·7–11·9) | 7·0 (5·2–16·0) | 9·7 (7·4–15·9) | 14·0 (11·0–15·0) | 14·0 (12·4–15·1) | 15·0 (13·0–15·0) | 15·0 (14·0–15·4) |
| **Pulmonary artery occlusion pressure (PAOP)** (mmHg) | | | | | | | |
| Before iNO | 11 | 17 | 10 | 12 | 8 | 6 | 12 |
| PAOP during iNO application | | | | | | | |
| Minimum | 8 | 13 | 5 | 9 | 3 | 2 | 8 |
| Maximum | 18 | 21 | 13 | 16 | 11 | 9 | 14 |
| Median (IQR) | 13 (12–14) | 16·5 (14·8–18·3) | 10 (9–12) | 13·5 (13–15) | 10 (9–10·5) | 7 (4–8) | 11 (11–12) |
| **PAOP after iNO cessation** | | | | | | | |
| Minimum | 7 | 6 | 7 | 11 | N/A | 2 | 6 |
| Maximum | 7 | 11 | 10 | 13 | N/A | 9 | 11 |
| Median (IQR) | 7 (7–7) | 10 (6·5–10·5) | 9 (7–..) | 12 (11·5–13) | N/A | 8 (3·5–8·8) | 10 (6–..) |
| **Cardiac index during iNO application** | | |  |  |  | | |
| Minimum | 3·9 | 2·8 | 2·7 | 2·9 | 2,9 | 2·0 | 2·8 |
| Maximum | 6·2 | 4·3 | 4·6 | 4·2 | 4,2 | 2·9 | 5·9 |
| Median (IQR) | 5·3 (4·5–5·6) | 3·6 (3·1–4·1) | 2·9 (2·8–3·1) | 3·6 (3·2–3·7) | 3.4 (3·3–3·8) | 2·2 (2·2–2·3) | 3·4 (3·2–5·2) |
| **Cardiac output during iNO application (L/min)** | | |  |  |  |  |  |
| Minimum | 3·7 | 4·4 | 4·5 | 5·2 | 5·1 | 3·0 | 5·6 |
| Maximum | 11·3 | 6·8 | 7·6 | 7·6 | 7·4 | 4·2 | 11·8 |
| Median (IQR) | 9·2 (7·6–9·8) | 5·9 (4·9–6·4) | 4·8 (4·6–5·1) | 6·5 (5·8–6·7) | 6·0 (5·8–6·7) | 3·2 (3·2–3·4) | 6·8 (6·4–10·6) |
| **Creatinine** (µmol/l) |  |  |  |  |  |  |  |
| Before iNO | 57 | 44 | 71 | 47 | 50 | 51 | 67 |
| Maximum during iNO | 54 | 41 | 60 | .. | 50 | 52 | 67 |
| **INR** |  |  |  |  |  |  |  |
| Before iNO | <1 | <1 | 1·00 | 1·06 | <1 | <1 | <1 |
| Maximum during iNO | <1 | <1 | <1 | 1·01 | <1 | <1 | 1·02 |
| **aPTT** (sec) |  |  |  |  |  |  |  |
| Before iNO | 27·4 | 30·1 | 26·2 | .. | 29·2 | .. | 26 |
| Maximum during iNO | 28·2 | 32·1 | 24·2 | 27·1 | 31·8 | 34·1 | 31·5 |
| **SvO_2_** (%) during iNO application | |  |  |  |  |  |  |
| Minimum | 75 | 77 | 67 | 66 | 66 | 61 | 65 |
| Maximum | 83 | 82 | 71 | 83 | 83 | 72 | 82 |
| Median (IQR) | 76 (76–78) | 80 (79–81) | 69 (68–70) | 72 (70·8–75) | 78 (77·5–80) | 70 (68·8–71) | 71 (69·5–79·5) |
| **PaO_2_** (mmHg) during iNO application | |  |  |  |  |  |  |
| Minimum | 141 | 89 | 144 | 96 | 124 | 140 | 114 |
| Maximum | 181 | 130 | 153 | 118 | 209 | 157 | 139 |
| Median (IQR) | 164 (145–181) | 117 (117–130) | 148 (145·5–152) | 110 (108–116) | 170 (140·8–186·5) | 142 (140·5–151·5) | 119 (115–135) |
